# *Pluteus insidiosus* Complex, Four New Species Described and *Pluteus reisneri* Resurrected

**DOI:** 10.3390/jof8060623

**Published:** 2022-06-10

**Authors:** Hana Ševčíková, Giuliano Ferisin, Ekaterina Malysheva, Alfredo Justo, Jacob Heilmann-Clausen, Egon Horak, Lyudmila Kalinina, Oğuzhan Kaygusuz, Henning Knudsen, Nelson Menolli, Pierre-Arthur Moreau, Guillermo Muñoz González, Irja Saar, İbrahim Türkekul, Francesco Dovana

**Affiliations:** 1 Department of Botany, Moravian Museum, Zelný trh 6, CZ-659 37 Brno, Czech Republic; hsevcikova@mzm.cz; 2 Associazione Micologica Bassa Friulana, Via Vespucci 7, 33052 Cervignano del Friuli, Italy; gferisin@gmail.com; 3 Independent Researcher, 194021 St. Petersburg, Russia; ef.malysheva@gmail.com; 4 New Brunswick Museum, 277 Douglas Ave., Saint John, NB E2K 1E5, Canada; alfredo.justo@nbm-mnb.ca; 5 Center for Macroecology, Evolution and Climate, GLOBE Institute, University of Copenhagen, Universitetsparken 15, 2100 Copenhagen, Denmark; jheilmann-clausen@sund.ku.dk; 6 Independent Researcher, AT-6020 Innsbruck, Austria; sporax@gmx.net; 7 Independent Researcher, 190005 St. Petersburg, Russia; smillak@gmail.com; 8 Department of Plant and Animal Production, Atabey Vocational School, Isparta University of Applied Sciences, Isparta 32670, Turkey; okaygusuz03@gmail.com; 9 Independent Researcher, 1825 Copenhagen, Denmark; hknudsen23@outlook.dk; 10 IFungiLab, Subarea of Biology (SAB), Department of Natural Sciences and Mathematics (DCM), Câmpus São Paulo (SPO), Federal Institute of Education, Science and Technology of São Paulo (IFSP), Rua Pedro Vicente, 625, Sao Paulo 01109-010, Brazil; menollijr@yahoo.com.br; 11 Faculté de Pharmacie, University of Lille, ULR 4515—LGCgE, F-59000 Lille, France; pierre-arthur.moreau@univ-lille.fr; 12 Independent Researcher, 50650 Gallur, Spain; guillermomunoz1981@gmail.com; 13 Institute of Ecology and Earth Sciences, University of Tartu, J. Liivi 2, 50409 Tartu, Estonia; irja.saar@ut.ee; 14 Department of Biology, Faculty of Science and Arts, Gaziosmanpaşa University, Tokat 60010, Turkey; turkoibrahim@yahoo.com; 15 Independent Researcher, 15029 Solero, Italy

**Keywords:** Agaricales, new species, Palaearctic, Pluteaceae, type study

## Abstract

We studied the taxonomy of *Pluteus insidiosus* and similar species using morphological and molecular (nrITS, *TEF1-α*) data, including a detailed study of the type collection of *P. insidiosus*. Based on our results, we recognize five species in this group: *P. insidiosus* sensu stricto and four other taxa: *P. assimilatus*; *P. farensis*; *P. flavostipitatus;* and *P. pseudoinsidiosus;* described here as new. All these taxa are distinct from each other based on molecular data, but some of them are semi-cryptic based on morphology and co-occur in the Palaearctic region. An additional molecular lineage, phylogenetically separates from the *P. insidiosus* complex, but with many morphological similarities, was recognized in the molecular phylogenies. Based on the revision of available type collections, the name *Pluteus reisneri* Velen., was adopted for this Clade. *Pluteus reisneri* was validly published in 1921, but it has barely been used since its original description. A modern epitype, with molecular data, was selected for *P. reisneri*.

## 1. Introduction

The agaricoid genus *Pluteus* Fr. belongs to the family *Pluteaceae* Kotl. and Pouzar [1] and is characterized by producing basidiomata with free lamellae and without volva or universal veil, a pinkish spore print, smooth thick-walled basidiospores, an inverse hymenophoral trama, the presence of cheilocystidia and often also pleurocystidia [2,3]. It has an estimated diversity of ca. 500 species occurring in boreal, temperate, tropical, and austral forest ecosystems and all transitional areas [4]. The genus has been traditionally subdivided in sections and subsections according to the morphology of the pleurocystidia and the morphology and organization of the pileipellis elements [5]. One of these infrageneric groups, *Pluteus* sect. *Celluloderma* Fayod, was traditionally divided into two subsections based on morphology [6,7]. *Pluteus* subsect. *Eucellulodermini* Singer was characterized by a hymeniderm or epithelial pileipellis consisting of clavate to sphaeropedunculate elements [2,6,7]. *Pluteus* subsect. *Mixtini* Singer was characterized by a similar pileipellis structure but with the presence of additional elongated elements [2,6,7]. However, early phylogenetic works on *Pluteus* showed that species morphologically assignable to either subsection did not form monophyletic groups [8,9,10,11], although the presence or absence of elongate pileipellis elements is still a valuable taxonomic character at the species-level.

*Pluteus insidiosus* Vellinga and Schreurs was described from the Netherlands as resembling *P. thomsonii* (Berk and Broome) Dennis, especially as a result of the rostrate cystidia, but differing in the absence of elongated elements in the pileipellis [12]. Due to this key morphological difference, *P. insidiosus* was placed in the *Pluteus* subsect. *Eucellulodermini,* whilst *P. thomsonii* was placed in the *Pluteus* subsect. *Mixtini* [12]. Previous phylogenies showed that this segregation lacked phylogenetic support, and that collections identified as *P. insidiosus* and *P. thomsonii* formed distinct branches within the “thomsonii clade” in the *Pluteus* sect. *Celluloderma* [9,13]. The group is however still not fully resolved, and phylogenetic reassessments suggest that several additional species might be hiding under those names [5,8,9,14]. Morphologically similar species were described in recent years, e.g., *P. diverticulatus* Corriol [15], and older names that have not been used by modern authors and/or are considered synonyms of *P. thomsonii* [3] are in need of re-evaluation: *P. reisneri* Velen. [16], *P. pilatii* Velen. [17], *P. terrestris* Velen. [18], *P. cinereus* Quél. [19], *P. cinereus* var. *venosus* Vacek [20] and *P. cinereus* var. *evenosus* Kühner [21].

The aim of this paper is to study taxonomy and phylogeny of *P. insidiosus* and phylogenetically related species characterized by a pileipellis predominantly made of sphaeropedunculate to clavate elements, including the species present in the temperate and boreal areas of Eurasia, which approximately corresponds to the Palaearctic region defined by Kreft and Jetz [22].

The main outcomes of our taxonomic revision are: (i) redescription of *P. insidiosus* based on the re-examination of the type collection, and analysis of molecular data derived from it; (ii) description of four new species closely related to *P. insidiosus*: *P. assimilatus, P. farensis, P. flavostipitatus* and *P. pseudoinsidiosus*; and (iii) resurrection of the name *P. reisneri* for one of the species recognized in the phylogenetic analyses, with the selection of a modern epitype to guide the application of this name.

## 2. Materials and Methods

### 2.1. Morphology

Color abbreviations follow the RAL Design color range system (https://www.ralcolorchart.com/ral-design, accessed on 3 April 2022) [23]; herbarium abbreviations are according to Thiers [24]; FG = G. Ferisin’s personal herbarium. Microscopic features were described from dried material mounted in 10% KOH and Congo Red with a magnification of 600× and 1000×. Abbreviations: Lav mean of basidiospore length; Wav mean of basidiospore width; Q = quotient of length and width in any one basidiospore; Q* = mean of basidiospore Q values. The following abbreviations are used: L = number of lamellae reaching the stipe, l = number of lamellulae between each pair of lamellae; the notation [X, Y, Z] indicates that measurements were made on X spores, in Y samples from Z collections

Macroscopic descriptions of newly collected specimens are based on fresh basidiomata, except of dry specimens of *Pluteus insidiosus* and *P. reisneri* holotypes.

### 2.2. Molecular Phylogeny

#### 2.2.1. DNA Extraction, Amplification, Sequencing and Sequence Alignment

For DNA extraction, small fragments of dried basidiomata were used. Collections deposited on the herbaria L, BRNM and LIP were performed by M. Sochor and followed the molecular methods described by Ševčíková et al. [25]. For collections on LE, the procedure of DNA extraction completely corresponded to the manufacturing protocol of the Phytosorb Kit (ZAO Syntol), and for collections on GDOR the DNA was extracted with NaOH following the procedures reported in Dovana et al. [26]. The following primers were used for amplification and sequencing: ITS1F-ITS4/ITS4B [27,28] for the internal transcribed spacer (nrITS: nrITS1-5.8S-nrITS2) fragment, and EF1-983F and EF1-1567R for part of the translation elongation factor 1-alpha (*TEF1-α*) [29]. PCR products were purified applying the GeneJET Gel Extraction Kit (Thermo Scientific, Thermo Fisher Scientific Inc., Waltham, MA, USA). Raw data were edited and assembled in MEGA 10 [30].

#### 2.2.2. Phylogenetic Analyses

We assembled an nrITS dataset of all available sequences phylogenetically close to *P. insidiosus* and *P. thomsonii* (“thomsonii clade” in Menolli et al. [9]). This includes 24 newly generated nrITS sequences for this study, and 35 sequences generated in previous studies (see Table 1; [5,9,14,31,32,33,34,35,36]) or available in public databases and biodiversity repositories (GenBank, UNITE, iNaturalist; see Table 1). A total of 59 nrITS sequences were used in the final dataset, including voucher-based and environmental sequences. We assembled a *TEF1-α* dataset of 17 sequences, 16 of them newly generated for this study and an additional sequence previously available in GenBank (see Table 1, [36]). In all datasets we included sequences of *P. phlebophorus* and *P. rugosidiscus* as outgroup taxa, based on previous phylogenetic work on *Pluteus* [8,9]. Sequences were aligned using MAFFT version 7 [37] and the strategy FFT-NS-i. The alignment was inspected and manually corrected in AliView [38]. No topological conflicts were detected in the phylogenetic analyses of the nrITS and *TEF1-α* datasets (detailed below), so a combined dataset was created by concatenating the nrITS and *TEF1-α* matrices.

For all three datasets (nrITS, *TEF1-*α and nrITS+*TEF1-*α) three separate phylogenetic analyses were run: (i) maximum likelihood (ML) analyses using RAxML 8.2.10 [39] under a GTRGAMMAI model with 1000 rapid bootstrap (BS) replicates; (ii) Bayesian inference (BI) analyses using MrBayes 3.2.7 [40] for 10 million generations under a GTRGAMMAI model with four chains, and trees sampled every 1000 generations. The initial burn-in phase was set to 2.5 million generations, and this value was confirmed to be adequate by checking the graphic representation of the likelihood scores of the sampled trees. Additionally, we also confirmed that the standard deviation of split frequencies was < 0.05, and that PRSF values were close to one, as detailed in Ronquist et al. [41]. All analyses were run using resources at the CIPRES Science Gateway [42]. In order to best understand the relationships between the different species within the Clade I, an Intra- and Inter-specific patristic distances for each region were calculated in Geneious R11 [43] using RaxML 8.2.11 with GTR GAMMA model. Intra- and Inter-specific distances were represented by using boxplots drawn in Rstudio Version 1.1.453 using ggplot2 library [44].

## 3. Results

### 3.1. Phylogeny

The nrITS and *TEF1-α* datasets comprised 719 and 574 characters, respectively, and the final combined nrITS + *TEF1*-α dataset consisted of 59 sequences and a total of 1293 characters (gaps included). All individual and combined datasets and their respective ML and BI trees have been deposited at TreeBASE (S28792).

**Figure 1:**
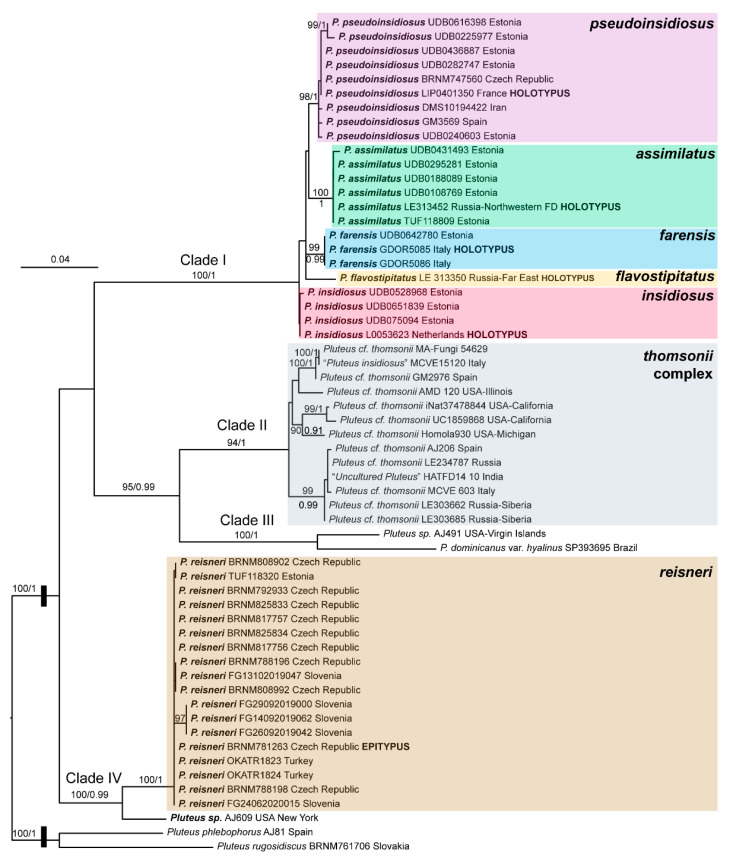
Best tree from the ML analysis of the nrITS + *TEF1-α* dataset. Bootstrap values ≥ 70% and posterior probabilities ≥ 0.90 are indicated on or below the branches. The root length has been reduced to facilitate graphical representation.

There were no major differences in the overall topologies of the best tree from the ML analysis and the consensus tree from the BI analysis. In Figure 1, we present the best tree from the ML analysis of the nrITS + *TEF1-*α dataset, with bootstrap values ≥ 70% and posterior probabilities ≥ 0.90.

Four distinct clades can be recognized in the analyses:

(i) **Clade I** includes *P. insidiosus* and the newly described *P. pseudoinsidiosus*, *P. assimilatus*, *P. farensis* and *P. flavostipitatus*. All taxa, except *P. insidiosus* received high support in the ML and BI analyses. None of the sister-taxa relationships received significant support in any of the analyses;

(ii) **Clade II** includes thirteen sequences: eleven sampled collections assignable to *P. thomsonii* in the broad sense; one of the public sequences in GenBank (coll. MCVE15120) has been originally named *P. insidiosus,* but it represents an incorrect identification and a second sequence (coll. HATFD14-10) reported as “Uncultured Pluteus”. This Clade includes five phylospecies that will be treated separately in a subsequent paper;

(iii) **Clade III** includes two tropical taxa, *P. dominicanus* var. *hyalinus* from Brazil and a likely undescribed species from the US Virgin Islands;

(iv) **Clade IV** includes *P. reisneri* and a likely undescribed species from the USA (New York).

Only the sister-clade relationship between Clades II and III received good support. Intra- and interspecific patristic distances between the different species within Clade I showed variable values within the different regions.

**Figure 2:**
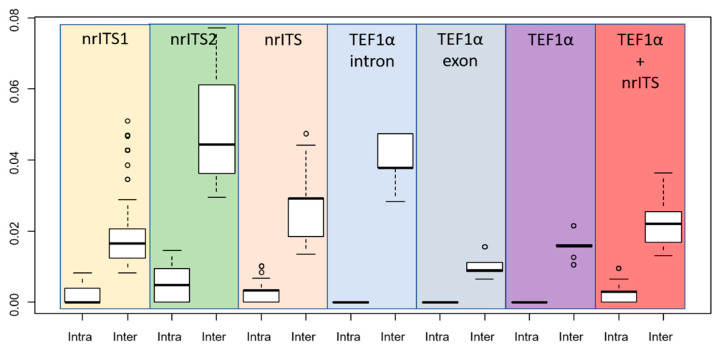
Boxplots illustrating variability of patristic distances between collections of *Pluteus* belonging to the Clade I. Intra- and interspecific variation comparison across: nrITS1, nrITS2, nrITS (nrITS1-5.8S-nrITS2), *TEF1-α*-intron, *TEF1-α*-exon, *TEF1-α*, nrITS + *TEF1-α*.

The boxplots of patristic distances of nrITS1, nrITS2, complete nrITS, translation elongation factor 1 alpha exon, intron and complete regions are reported in Figure 2. The nrITS intraspecific patristic distances ranged from 0.0000 to 0.0100, and interspecific distances from 0.0134 to 0.047. The nrITS2 was the most variable sub-region among those considered (intraspecific distances from 0.0000 to 0.0146 and interspecific distances from 0.0295 to 0.0772), highlighting it to be the best single region to separate species within the Clade I. nrITS1 intraspecific distances ranged from 0.0000 to 0.0082, and interspecific distances from 0.0082 to 0.0509, *P. insidiosus* showed hypervariability in the nrITS1 region, and its maximum intraspecific distance corresponded to minimum interspecific distances. *TEF1*-α interspecific patristic distances ranged from 0.0106 to 0.0216 (*TEF1*-α-exon: 0.0065 to 0.0156; *TEF1*-α-exons: 0.0282 to 0.0475).

### 3.2. Taxonomy

Here we present the descriptions of the six species phylogenetically and/or morphologically related to *Pluteus insidiosus* currently known to occur in the Palaearctic region. Generally, there are only a few distinct morphological or ecological differences between the treated taxa, and in many cases, identification of individual basidiomata without molecular data will prove difficult. Despite these difficulties, we do interpret these phylogenetic lineages as separate species, and therefore they have to be described and named. Without a correct understanding of the natural history of species in the *Pluteus insidiosus* complex and a transparent taxonomy and nomenclature, it will be impossible to obtain more accurate data about the distribution, ecology, morphology and conservation status of these taxa. Regional endemics in this group need further studies to establish their possible conservation status. Morphological features are discussed below, and the differences are presented in the key.

**Figure 3:**
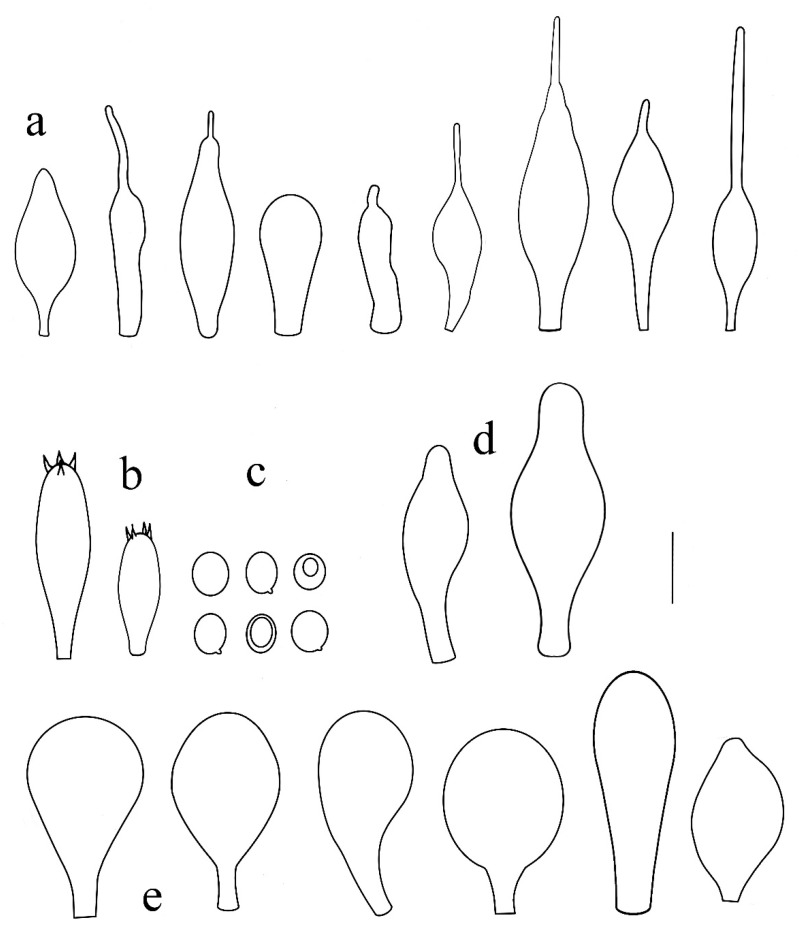
*Pluteusinsidiosus* holotype L0053623. (**a**) cheilocystidia, (**b**) basidia, (**c**) spores, (**d**) pleurocystidia, (**e**) pileipellis elements. Scale bar 10 μm.

Notes: Vellinga and Schreurs [12] described the cheilocystidia of *P. insidiosus* as being not crowded, but later, Vellinga [3] mentioned the lamellar edge as being sterile. Our examination of the holotype showed that in some places, cheilocystidia form clusters rendering the lamellar sterile, while in other portions, the lamellar edge was heterogeneous, with basidioles, and in some places even basidia intermixes with the cheilocystidia. However, these fertile sites were not predominant, and it remains uncertain if the nature of the lamellar edge is a stable separating character. Although the phylogenetic analysis presented in Figure 1 does not statistically support the /P. insidiosus Clade, excluding the nrITS1 region and the *TEF1-*α introns from the previously used dataset, ML analysis supports the /P. insidiosus Clade (ML bootstrap = 76%) (tree reported in Supplementary Figure S1). Within Clade I, *P. insidiosus* is characterized by -TC-deletion in nrITS1, -T-insertion in nrITS2 and a non-silent mutation in *TEF1-α-exon* (GTC/Valine instead of GCC/Alanine).

*Pluteus insidiosus* Vellinga and Schreurs (Figure 3)

Vellinga, E.C.; Schreurs, J. 1985. Notulae ad Floram Agaricinam Neerlandicam—VIIl. Persoonia. 12(4): 337–373

Type L0053623

Protologue [12]: Pileus 25–40 mm, planoconvex, applanate, with low umbo, slightly hygrophanous, when moist very dark brown to black in centre, pallescent towards margin to dark brown or brown (Munsell 7.5 YR 2/1–3/3), with translucently striate margin, on drying pallescent to brown (Munsell 7.5 YR 4/4), in centre venulose or smooth. Lamellae (L = 48–72/1 = 0–3) fairly crowded, free, slightly ventricose, up to 5 mm broad, first pink, later incarnate, sordid or brownish pink, with an even concolorous edge. Stipe 35–45 mm, cylindrical or slightly broadening downwards, solid, whitish or silverish grey, innately fibrillose, glabrous and shiny. Context in pileus hygrophanous, when moist grey, pallescent on drying to whitish; in stipe concolourous with surface. Smell absent or bitterish; taste absent or slightly unpleasant.

Holotype revision: basidiospores [60/1/1] (5.6–)6.1–7.9(–8.5) × (5.1–)5.5–7.1(–7.4) µm, Lav = 7.0 µm, Wav = 6.1 µm, Q = (1.0–)1.05–1.25(–1.4), Q* = 1.15, subglobose to broadly ellipsoid, a few ellipsoid or globose. Basidia (20–)27–38(–45) × (5.5–)7.2–9.5(–11.0) µm, mostly clavate, four-spored. Pleurocystidia was very rare, 45–56(–75) × 19–28(–40) µm, utriform to broadly utriform or fusiform, thin-walled and colorless. Lamellar edge heterogenous, in some parts fertile. Cheilocystidia not crowded in some parts of lamellae, in other places in clusters, (21–)29–43(–54) × (6.0–)6.8–13.5(–19.5) µm, clavate to narrowly subutriform, rarely subfusiform to fusiform, some with narrow 1–40 × 1–3 µm long rostrum at apex, colorless. Pileipellis a hymeniderm of sphaeropedunculate to narrowly clavate elements (25–)31–57(–64) × (12–)16–33(–41) µm, with brown vacuolar pigment. Stipitipellis a cutis of cylindrical, colorless, 4–10 µm wide hyphae; caulocystidia absent. Clamp connections absent at all septa.

Habitat. Solitary on stumps of deciduous trees (*Alnus* sp., *Fagus* sp.), *Alnetum* sp. and *Fagetum nudum*; August–October.

Collections examined: NETHERLANDS, prov. Gelderland: Buren, loam-pits, 22 August 1979, leg. M. E. Noordeloos 983 (coll. J. Schreurs 315, holotype, L0053623); ibidem, 22 August 1979, M. E. Noordeloos s.n., (coll. J. Schreurs 316 L).

**Figure 4:**
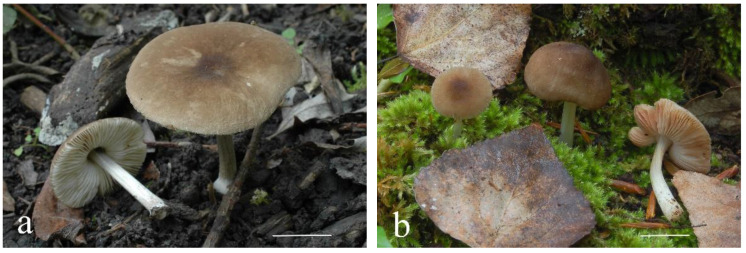
*Pluteus pseudoinsidiosus* basidiomata in situ: (**a**) holotype LIP0401350; (**b**) *P. pseudoinsidiosus* basidiomata in situ, GM3569.

**Figure 5:**
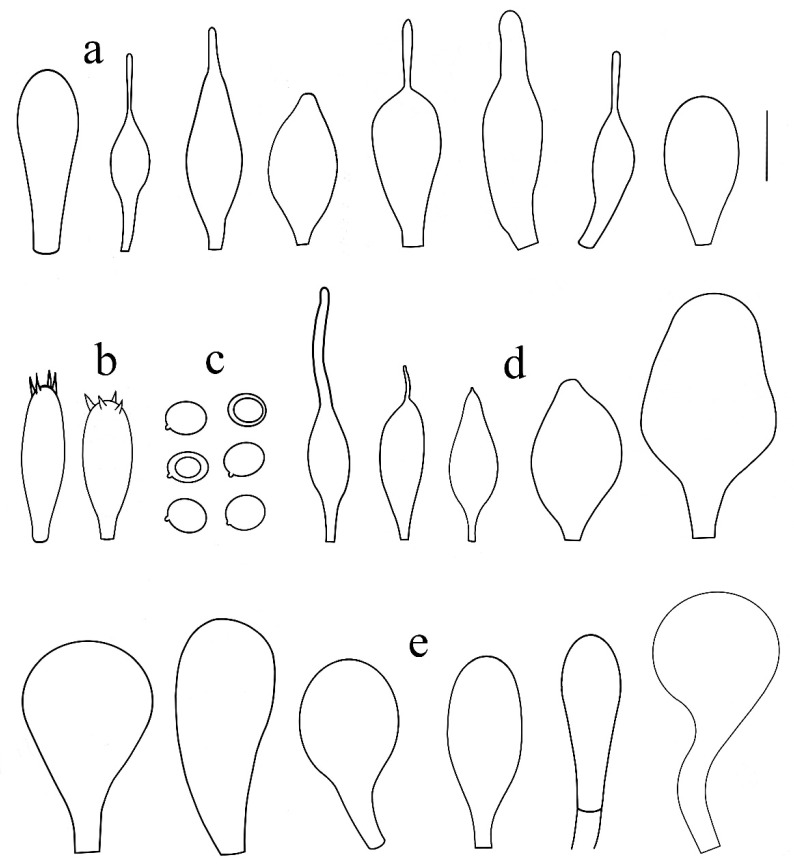
*Pluteus pseudoinsidiosus* holotype LIP0401350: (**a**) cheilocystidia; (**b**) basidia; (**c**) spores; (**d**) pleurocystidia; (**e**) pileipellis elements. Scale bar 20 μm.

*Pluteus pseudoinsidiosus* Ševčíková, Heilm.-Claus., Justo, P.-A. Moreau and G. Muñoz sp. nov. (Figure 4 and Figure 5)

MycoBank: MB843543

Etymology: —the specific epithet “*pseudoinsidiosus*” means similar to *Pluteus insidiosus*.

Diagnosis: —differs from *Pluteus insidiosus* by its rare rostrate pleurocystidia, entirely sterile lamellar edge and distinctly different nrITS and *TEF1*-α-α sequences.

Holotype: —FRANCE: Nord, Santes, La Gite, Parc de la Deûle, 50.577N 2.968E, on rich organic soil, nitrophile place with *Salix* spp., 24 September 2017 leg. P.-A. Moreau (LIP0401350).

Pileus 15–33 mm diam., hemispherical, then convex, later plano-convex to applanate, with low or indistinct umbo, slightly to strongly hygrophanous, brown or olive-brown, warm tawny brown (RAL 070 40–60 30, RAL 075 60 20–30, RAL 075 50 20–40, RAL 075 40 20–30, RAL 80 30 20, RAL 80 60 30), darker in the center (RAL 070 30 20, RAL 075 20 10, RAL 070 20 10, RAL 095 20 10) with translucently striate margin sometimes only when wet, faintly rugulose at center. Lamellae (L = 48–72/1 = 0–2) free, crowded, slightly ventricose, up to four millimeters broad, whitish or pale gray when young, later pale pink with cream tinge, with an even concolorous edge. Stipe 20–42 × 1.5–3.0 (3.5) mm, cylindrical or slightly broadening toward base, solid, innately longitudinally fibrillose, glabrous or white-furfuraceous on the 1/4th lower part, shiny, whitish or silvery grey (RAL 075 92 05, RAL 080 90 05–10, RAL 90 80 10), sometimes with greenish blue tinge. Context thin, in pileus hygrophanous, when moist grey to greyish brown, pallescent on drying to whitish; in stipe concolorous with surface or greyish brown. Smell and taste indistinct.

Basidiospores [160/4/6] (4.8–)5.0–7.0(–7.2) × 4.3–5.5(–6.0) µm, Lav = 6.0 µm, Wav = 5.0 µm, Q = (1.0)1.1–1.3(–1.4), Q* = 1.19, subglobose to broadly ellipsoid, a few globose or ellipsoid. Basidia (20)25–38(42) × (6)7–10(11) µm, clavate, 4-spored, rarely 2 or 1-spored. Pleurocystidia rare, (20–)25–40(–44) × 7–18(–22) µm, narrowly clavate to clavate, narrowly subfusiform or subutriform, or irregular, some with narrow, needle-like, 2–40 μm long rostrum at apex; rarely 40–55 × 20–33 µm broadly subutriform to subfusiform with broad apex and short pedicel, thin-walled, rarely slightly wider up to 0.5 µm, colorless. Lamellar edge sterile, cheilocystidia (24–)26–45(–50) × 7–15(–25) µm, quite variable, clavate to narrowly subutriform, some with narrow, needle-like, 1–40 μm long rostrum at apex, colorless, thin-walled, very rarely slightly thick-walled. Pileipellis a hymeniderm of sphaeropedunculate to narrowly clavate elements, (22–)35–58(–62) × (8.0–)15–31(–33) µm, with olive-brown or brown vacuolar pigment. Stipitipellis a cutis of cylindrical, 4.0–11.0 µm wide hyphae, colourless or with indistinct grey tinge; caulocystidia absent or very rare near the stipe base, 20–23 × 5.0–10.0 μm clavate to subfusiform with or without rostrum up to 11.0 × 2.5 μm. Clamp connections absent at all septa.

Habitat. Fagetum, floodplain forest (*Fraxinus angustifolia*, *Carpinus betulus*, *Quercus robur*, *Acer campestre* and *Ulmus* spp.) and riverside forest interspersed with *Quercus ilex*. Solitary on stumps and fallen trunks of deciduous trees or on soil. May–October.

Additional specimens examined: CZECH REPUBLIC. Lanžhot, Ranšpurk Nature Reserve, floodplain forest—*Fraxinus angustifolia*, *Carpinus betulus*, *Quercus robur*, *Acer campestre* and *Ulmus* spp., deciduous stump covered with moss, 48.677N 16.947E, 24 May 2013 leg. H. Ševčíková (BRNM747560); IRAN. Golestan, Gorgon, +/−virgin beech forest at 950 m elevation, on the wood debris zone underneath a big fallen log of *Fagus orientalis*, 3 October 2016, leg. Jacob Heilmann-Clausen and Claus Bässler (DMS-10194422; collected as *Pluteus cyanopus*); SPAIN. Tudelilla, on a decayed stump of *Populus nigra*, in a small riverside forest interspersed with *Quercus ilex*, 42.2927N 2.1504W, 16 May 2020 leg. G. Muñoz González (GM3569).

Notes: Based on our current knowledge, *Pluteus pseudoinsidiosus* is macroscopically almost indistinguishable from *P. insidiosus*, except for a somewhat paler pileus with ochraceous or olivaceous tones, which may however not be a stable feature; as well as a greenish blue stipe if present. Microscopically, these two taxa differ only by inconspicuous features. Basidiospores of *P. pseudoinsidiosus* are smaller than those of *P. insidiosus* and many pleurocystidia of *P. pseudoinsidiosus* have rostrum, which has never been observed in *P. insidiosus*. However, pleurocystidia are scarce and difficult to find in both taxa. They might even be absent in some collections of both species (DMS-10194422, Vellinga [3]). The sterile lamellar edge seems to be another distinguishing feature, but a stability of this feature is uncertain. Based on known collections, *P. pseudoinsidiosus* seems to have a more southern distribution than *P. insidiosus*, but the sample size is too small to judge if this tendency is reflecting a real difference. Further experience with both taxa is needed, and at present these species may be regarded as cryptic and only separable by sequence data. Basidiomata of *P. pseudoinsidiosus* with a bluish stipe may resemble *Pluteus cyanopus* Quél. However, by its protologue [45], this species has a black or purplish pruinose pileus, greyish or lilac lamellae and basidiospores about 6 µm. Vellinga [3] interpreted this species based on recent collections with a bluish stipe by having a brown pileus, (narrowly) utriform to pedunculate and ovoid-conical pleurocystidia and narrowly utriform to ovoid cheilocystidia without rostrum. A discussion on the true identity of *Pluteus cyanopus* falls out of the scope of the present paper, but it will be discussed in another prepared article about the taxa placed in the /cinereofuscus and /phlebophorus clades sensu Menolli et al. [9] and Malysheva et al. [14]. *Pluteus phaeocyanopus,* Minnis and Sundb., also has a bluish stipe, but has larger basidiospores (6.2–8.4 × 5.7–7.9 µm), (narrowly) lageniform pleurocystidia with pedicel, long neck and obtuse apex with brown intracellular pigment and subglobose to pyriform cheilocystidia with brown intracellular pigment [46]. This species is known from Western North America (San Francisco, CA, USA), and molecular data based on Californian collections show that this species belong in the/cinereofuscus Clade, not related with the *P. insidiosus* complex (Data not shown).

**Figure 6:**
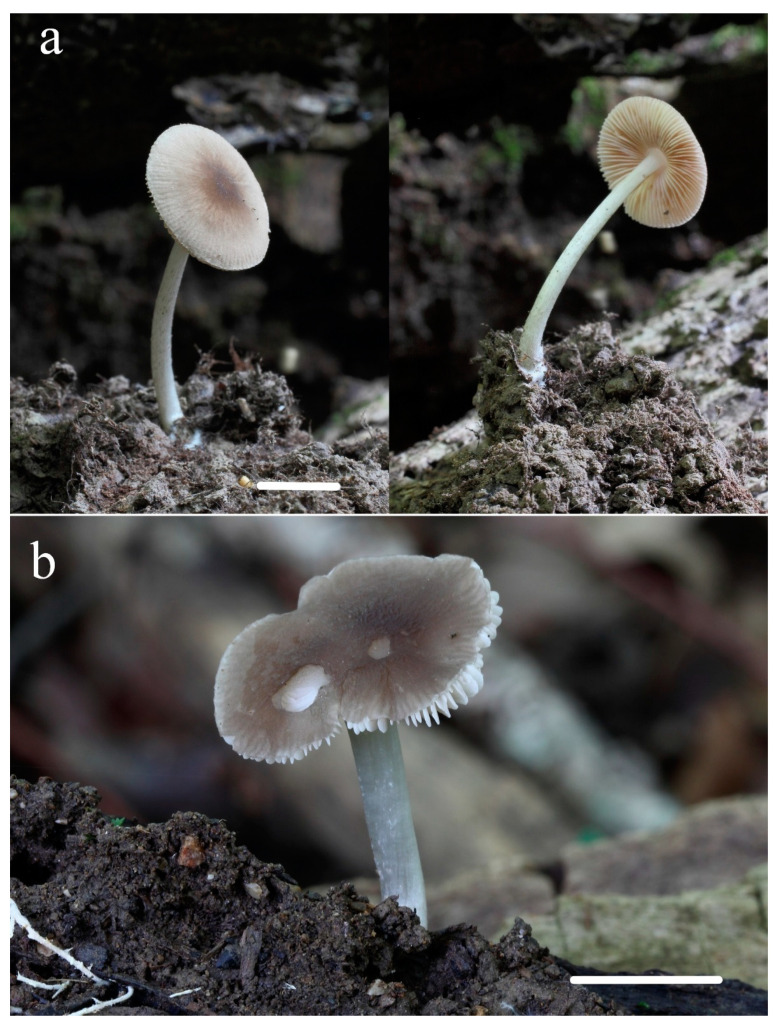
*Pluteusfarensis* basidiomata in situ: (**a**) GDOR 5086; (**b**) GDOR 5085 (holotype).

**Figure 7:**
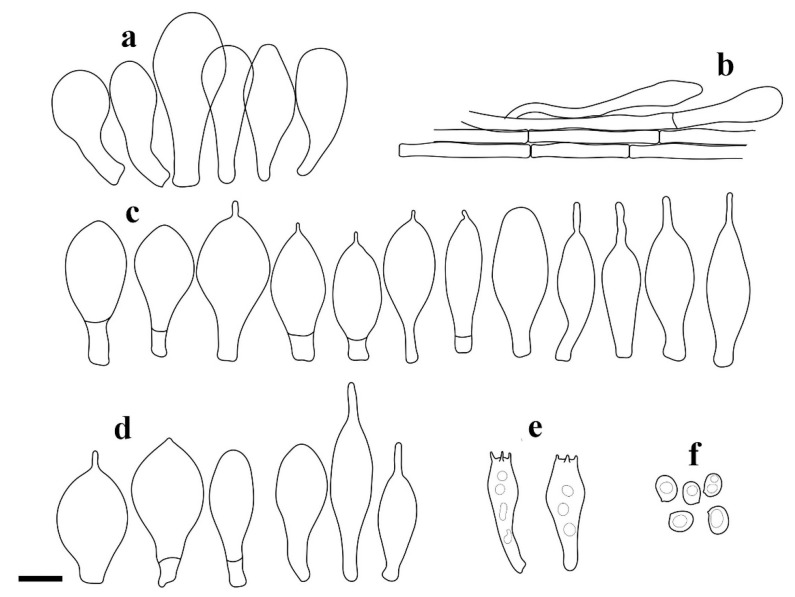
*Pluteusfarensis* holotype GDOR 5085: (**a**) pileipellis elements; (**b**) caulocystidia; (**c**) cheilocystidia; (**d**) pleurocystidia; (**e**) basidia; (**f**) spores. Scale bar 10 μm.

*Pluteus farensis* Ferisin and Dovana sp. nov. (Figure 6 and Figure 7)

MycoBank: MB843560

Etymology: named from “Fara”, a historical name of the city Farra d’Isonzo, the place where the holotype was found.

Diagnosis: differs from *Pluteus insidiosus* by its smaller basidiospores, cheilocystidia mucronate or rostrate with characteristic short apex, pleurocystidia scattered similar to cheilocystidia, lamellar edge fertile and different nrITS sequence.

Holotype: ITALY, Farra d’Isonzo, Soca (Isonzo) River, 45.903N 13.540E, 7 July 2018, leg. G. Ferisin (GDOR 5085).

Pileus 15–22 mm diam., initially convex, then expanded to applanate, without umbo, brown (RAL 070 30 20, RAL 070 40 20) in center, light brown towards the margin (RAL 070 60 20) becoming grey-brown with age (RAL 060 60 05); hygrophanous, striate up to half of radius. Surface opaque, velvety or glabrous, weakly to strong venose at the center. Lamellae (L = 40–44/1 = 0–2) moderately crowded, free, slightly ventricose up to four millimeters broad, initially whitish later pink with whitish flocculose edge. Stipe 25–32 × 1.5–2.0 mm, cylindrical usually broadened towards base, pubescent and striate lengthwise, whitish to light grey. Smell and taste not distinctive. Context white.

Basidiospores [60/2/2], (5.6–) 5.8–6.5(–7.1) × (4.5–)4.9–5.6(–6.0) µm, Lav = 6.1 µm, Wav = 5.2 µm, Q = (1.02–) 1.11–1.25(–1.30) Q* = 1.18; globose to subglobose rarely broadly ellipsoid, thick-walled, non-amyloid, cyanophilous. Basidia 30–35 × 8.0–10.0 µm, clavate, four-spored. Cheilocystidia 30–55 × 9–17 µm, rather numerous or scarce mixed with basidia, clavate to broadly clavate mucronate or with up to 12 µm rostrate apex, thin walled. Pleurocystidia scattered, similar to cheilocystidia. Pileipellis a hymeniderm made up of clavate elements, very rare mucronate, 30–52 × 8–18 µm, with light brown intracellular pigment. Stipitipellis a cutis of hyaline or light brown hyphae, rarely with oily contents, 4.0–10.0 µm wide. Caulocystidia only in the upper part of the stipe very close to the lamellae, clavate; 35–50 × 10–18 µm, in small clusters in the apical part of the stipe, hyaline or with oily contents. Clamp connections absent at all septa.

Habitat and distribution: in the floodplain area located in the proximity of the Isonzo River, solitary, on soil on small buried woody shrubs in broad-leaved woods with *Fraxinus* spp. and *Salix* spp., fruiting from July to August.

Additional specimen examined: ITALY. Farra d’Isonzo, Soca (Isonzo) River, on soil, 45.903N 13.540E, 8 August 2015, leg. G. Ferisin, (GDOR 5086).

Notes: *Pluteus farensis* is characterized by having a brown opaque pileus surface, weakly to strongly venose at the center; a whitish to light grey pubescent stipe; cheilocystidia and pleurocystidia mucronate or with short rostrum at the apex, basidiospores mainly globose or subglobose and caulocystidia present only on the upper part of the stipe. *Pluteus pseudoinsidiosus* is macroscopically similar to P. farensis, but differs as the first has cheilocystidia with longer rostrum at the apex up to 40 µm and sterile lamella edges, although this last feature needs further investigation (see Notes on *P. pseudoinsidiosus*). *Pluteus flavostipitatus* is similar to *P. farensis* but mainly differs for its yellowish stipe, absence of caulocystidia and smaller basidiospore size (4.9–5.7(–6.1) × (4.3–)4.5–5.1(–5.8) µm). *Pluteus assimilatus* is distinguished from *P. farensis* by the presence of caulocystidia rather numerous over the entire stipe surface and pleuro- and cheilocystidia without rostrum. *Pluteus reisneri* is macroscopically similar to *P. farensis*, but differs in having abundant caulocystidia grouped in clusters on the lower part of stipe, pileipellis with some rostrate elements at the apex and the lack of the pleurocystidia.

**Figure 8:**
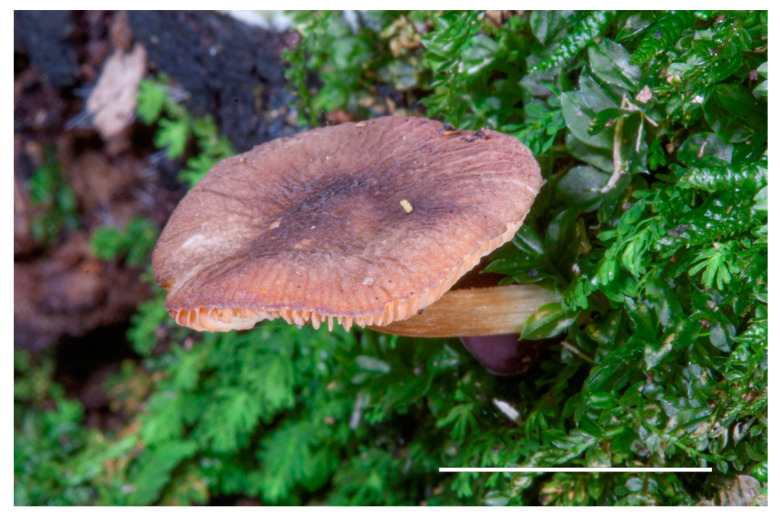
*Pluteus flavostipitatus* basidioma in situ, holotype LE 313350. Scale bar 10 mm.

**Figure 9:**
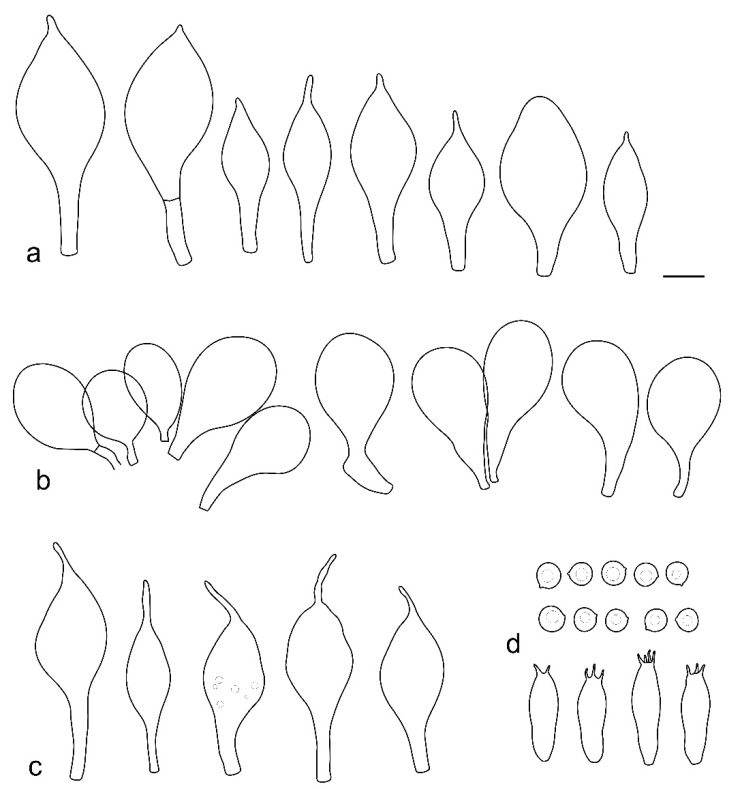
*Pluteus flavostipitatus* holotype LE 313350: (**a**) cheilocystidia; (**b**) pileipellis elements; (**c**) pleurocystidia; (**d**) basidia and spores. Scale bar 10 μm.

Notes: *Pluteus flavostipitatus* is characterized by rather small basidiomata with wrinkled pileus striate-sulcated at the margin, candle yellow and fibrillose stipe, rostrate pleuro- and cheilocystidia, rather small (5.0–6.2 × 4.5–5.7 µm) globose or subglobose basidiospores and the absence of caulocystidia. The most morphologically similar taxon, *P. pseudoinsidiosus*, differs from *P. flavostipitatus* in the whitish or silvery grey, rarely bluish stipe, long and prominent needle-like rostra of both pleuro- and cheilocystidia, slightly longer basidiospores (5.0–7.0 × 4.3–5.5 µm) and larger elements of pileipellis (35–58 × 15–31 µm). Differently from *P. flavostipitatus*, *P. insidiosus* possesses a larger, dark brown or black-brown pileus, whitish stipe, larger basidiospores and pleurocystidia that are never rostrate. *Pluteus farensis* is distinguished from *P. flavostipitatus* by the whitish stipe, the presence of caulocystidia and its different habitat.

*Pluteus flavostipitatus* E.F. Malysheva sp. nov. (Figure 8 and Figure 9)

MycoBank number: MB843561

Etymology: the name refers to yellowish colour of stipe.

Diagnosis: differs from *Pluteus pseudoinsidiosus* in sulcate margin of its pileus, yellowish stipe, short rostra of pleuro- and cheilocystidia, slightly smaller basidiospores (Lav × Wav = 5.3 × 4.9 µm) and different nrITS sequence.

Holotype: RUSSIA, Far Eastern Federal District, Primorye Territory, Land of the Leopard National Park, watershed of Ananyevka and Gryaznaya rivers, broadleaf forest, on moss covered trunk of deciduous tree, 43.39424 N, 131.52016 E, 2 September 2011, E.F. Malysheva (LE 313350).

Pileus 15 mm diam., convex to plano-convex, without umbo; slightly hygrophanous, striate and sulcate at margin; surface wrinkled, mat, laurel nut rusty brown (RAL 030 30 10), coffee brown (RAL 040 30 20) and wild brown (RAL 040 20 19). Lamellae free, rather distant, pink, with concolorous even edges. Stipe 17 × 1.5–2 mm, cylindrical without basal bulb, candle yellow (RAL 060-70-40), longitudinally fibrillose. Smell indistinct, taste not recorded.

Basidiospores [60/1/1] 4.9–5.7(–6.1) × (4.3–)4.5–5.1(–5.8) µm, Lav = 5.3 µm, Wav = 4.9 µm, Q = 1.0–1.2, Q* = 1.1, globose or subglobose, thick-walled. Basidia 23–30 × 6.2–7.0 µm, clavate with slightly constricted middle part, 4-spored. Pleurocystidia 36.5–51.0 × 11.0–17.0 µm, very scarce, narrowly to broadly clavate, with rostrum at apex, rostrum 8.5–13.0 × 1.5–2.3 µm, hyaline, thin-walled. Cheilocystidia 31.2–43.5(–54.5) × 10.0–21.5 µm, rather numerous, broadly clavate or fusiform, rarely utriform, with short refractive rostrum at apex, rostrum 3.0–8.0 × 1.3–2.4 µm, hyaline, thin-walled. Pileipellis a hymeniderm, made up of sphaeropedunculate, broadly clavate or utriform elements, 26.5–38.5 × 12.5–24.0 µm, with yellow-brown intracellular pigment, slightly thick-walled. Stipitipellis a cutis of cylindrical, hyaline, slightly thick-walled, 7.0–9.0 µm wide hyphae. Caulocystidia not seen. Clamp connections absent at all septa.

Habitat and distribution: In coniferous-broadleaf forest on moss covered trunk of deciduous tree. So far it is known only from type locality—Russia, Primorye Territory, Land of the Leopard National Park, mountain ridge. Vegetation along the watershed ridge is represented by complex coniferous-broadleaf forests of Manchurian type, where the dominant species are *Abies holophylla*, *Pinus koraiensis*, *Betula costata*, *Tilia amurensis* and *T. mandshurica*, as well as *Qurcus mongolica*. In the second vegetation layer maple (*Acer pseudosieboldianum*, *A. tegmentosum* and *A. mono*), *Betula schmidtii* and *Carpinus cordata* are dominated.

Collection examined: Holotype. RUSSIA, Far Eastern Federal District, Primorye Territory, Land of the Leopard National Park, watershed of Ananyevka and Gryaznaya rivers, mountain ridge, coniferous-broadleaf forest, broadleaf forest, on moss-covered trunk of deciduous tree, 43.39424 N, 131.52016 E, 2 September 2011, E.F. Malysheva (LE 313350).

**Figure 10:**
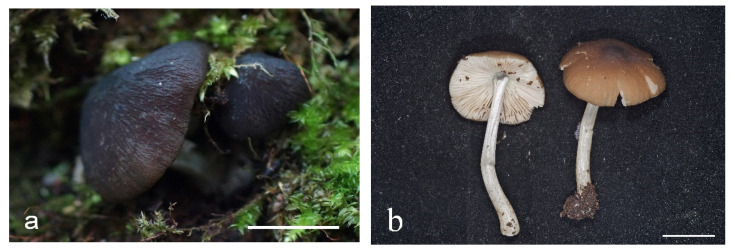
*Pluteus assimilatus* basidioma in situ: (**a**) LE 313452 (holotype); (**b**) TUF118809 (photo: Vello Liiv). Scale bar 10 mm.

**Figure 11:**
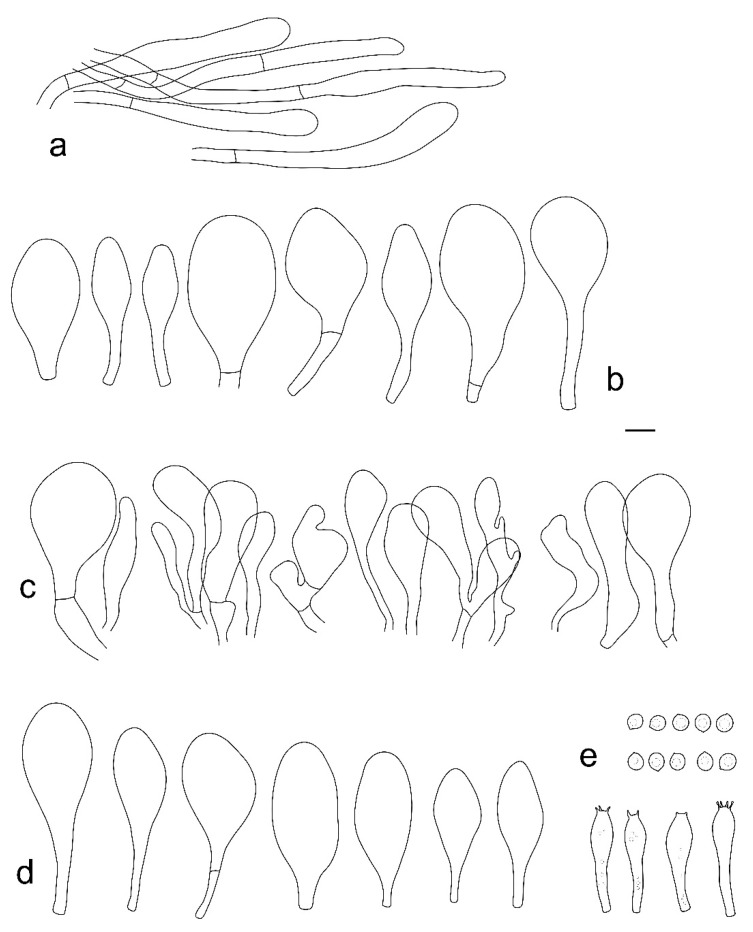
*Pluteus assimilatus* LE 313452 holotype: (**a**) caulocystidia; (**b**) cheilocystidia; (**c**) pileipellis elements; (**d**) pleurocystidia; (**e**) basidia and spores. Scale bar 10 μm.

Notes: *Pluteus assimilatus* shares several morphological features with *P. insidiosus*, having dark-colored pileus coupled with whitish or light-colored stipe, and broadly ellipsoid or subglobose basidiospores. However, the new species differs from the latter in absence of rostrate cheilocystidia, the presence of numerous caulocystidia, which have not been observed in *P. insidiosus*, larger cheilocystidia (41.5–71.0 × 16.5–32.5 µm vs 20–54 × 6–23 µm) and smaller basidiospores. Due to this characteristic (non-rostrate cystidia), the species is more similar to *P. nanus* than other members of the *P. insidiosus* complex, but differs in the smaller size of the basidiospores and the narrower pleurocystidia of a different shape.

*Pluteus assimilatus* E. F. Malysheva, L.B. Kalinina and I. Saar sp. nov. (Figure 10 and Figure 11)

MycoBank: MB843562

Etymology: the name emphasizes its morphological similarity with *Pluteus insidiosus*.

Diagnosis: differs from *Pluteus insidiosus* in smaller basidiospores (5.8–6.5 × 4.7–5.4 µm), larger cheilocystidia, the presence of numerous caulocystidia and different nrITS sequence.

Holotype: Russia, Northwestern Federal District, Leningrad Region, Lomonosovsky District, vicinity of Vilpovitsy village, 59.75621 N, 29.66867 E, slope covered with *Fraxinus excelsior*-*Acer platanoides* forest, on moss-covered trunk of deciduous tree, 13 July 2019, L.B. Kalinina (LE313452).

Pileus 15–35 mm diam., campanulate, convex to plano-convex, with or without umbo; not or slightly hygrophanous, not striate, but sometimes slightly sulcate at margin; slightly to strongly wrinkled all over the surface, from light oak brown (RAL 070 60 30) to coffee bean brown (RAL 060 40 20), night brown (RAL 050 20 16), deep brown (RAL 020 20 05), night red (RAL 020 20 10), or industrial black (RAL 060 20 05) at centre. Lamellae free, rather crowded, pink, with concolorous even edges. Stipe 15–30 × 2–4 mm, cylindrical with basal bulb, light grey (RAL 000 70 00), bleached white (RAL 270 90 05) or winter white (RAL 000 90 00), longitudinally fibrillose. Smell indistinct, taste not recorded.

Basidiospores [90/2/2] (5.4–)5.8–6.8(–7.5) × (4.4–)4.7–5.7(–6.8) µm, Lav = 6.1 µm, Wav = 5.5 µm, Q = 1.0–1.2(1.3); Q* = 1.2, broadly ellipsoid, or subglobose, some globose or ovoid, thick-walled. Basidia 25–36 × 6.5–10.5 µm, narrowly clavate or clavate, four-spored. Pleurocystidia 40.5–55.5(–71) × 15.0–23.5 µm, scarce, clavate, inflated-fusiform or oblong, with very short or predominantly without rostrum at apex, hyaline, thin-walled. Cheilocystidia 41.5–71.0 × 16.5–32.5 µm, numerous, broadly clavate, broadly utriform, inflated-fusiform, oblong or ellipsoid, without rostrum at apex, hyaline, thin-walled. Pileipellis a hymeniderm, made up of sphaeropedunculate, narrowly to broadly clavate, utriform or cylindrical elements, 33.0–56.5 × 11.5–19.0(–28.5) µm, some elements can be characterized as coralloid, i.e., they have one or more lateral short excrescences, with yellow-brown intracellular pigment, slightly thick-walled. Stipitipellis a cutis of cylindrical, hyaline, slightly thick-walled, 7.0–10.0 µm wide hyphae. Caulocystidia rather numerous, in bundles, narrowly clavate or cylindrical, 48–79 × 6–11 µm, with greyish brown intracellular pigment. Clamp connections absent at all septa.

Habitat and distribution: on trunks of broadleaved tree. The holotype was collected in the northwestern region of Russia, and the second collection studied was collected from Estonia. Thus, a presumable distribution area of the species may be limited to the territory of Northern Europe, but for precise knowledge additional finds are needed.

Additional collection examined: ESTONIA. Saare County, Kaarma Commune, Abruka Island, Abruka Nature Reserve, 58.15355 N, 22.49557 E, mixed deciduous forest (*Tilia cordata*, *Corylus avellana*, *Ulmus glabra*, *Quercus robur*, *Betula pendula*, *Picea abies*), on fallen trunk of deciduous tree, 20 September 2013, V. Liiv (TUF118809, as *Pluteus satur*).

In the nrITS + *TEF1-α* phylogenetic reconstruction (Figure 1), two collections of *P. assimilatus* formed one well-supported Clade that is sister (unsupported) to /*P. pseudoinsidiosus* Clade. All studied collections of *P. pseudoinsidiosus* differ from *P. assimilatus* in slightly larger basidiospores, rostrate pleuro- and cheilocystidia, and having shorter caulocystidia (20–23 × 5–7 μm) if they are present.

**Figure 12:**
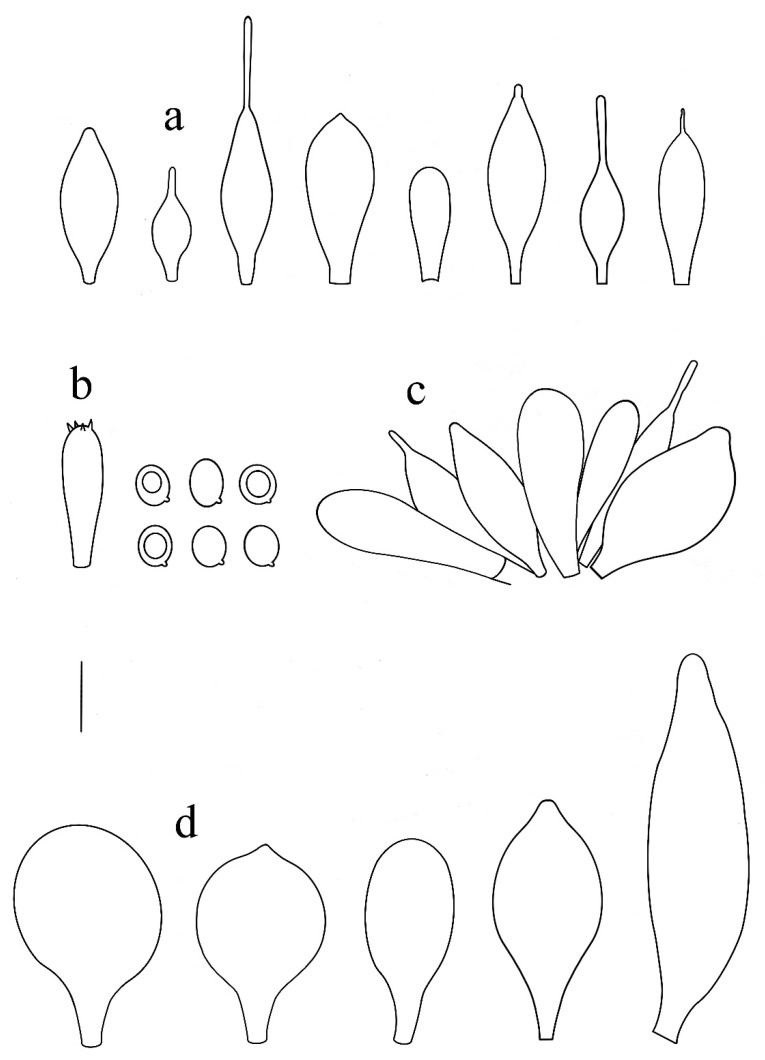
*Pluteus reisneri* holotype PRC, Velenovský herbarium, bottle 135: (**a**) cheilocystidia; (**b**) basidium and spores; (**c**) caulocystidia; (**d**) pileipellis elements. Scale bar 10 μm.

**Figure 13:**
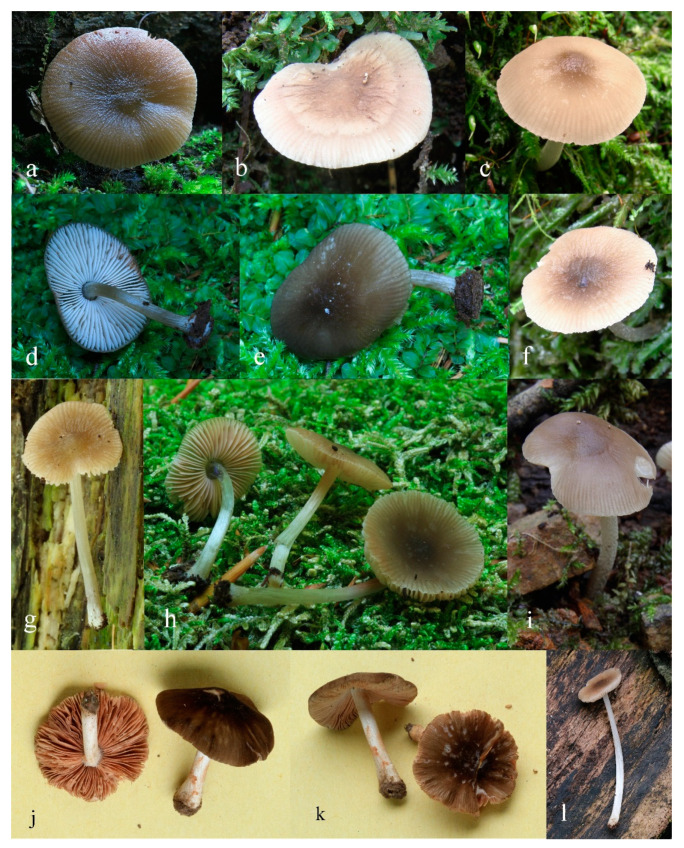
*Pluteus reisneri* basidiomata: (**a**) epitype BRNM781263; (**b**) FG24062020013; (**c**) FG14092019062; (**d**,**e**) BRNM781265; (**f**) FG13102019047; (**g**) BRNM808902; (**h**) BRNM817756; (**i**) FG26092019042; (**j**,**k**) BRNM825833; (**l**) OKA-TR1823.

**Figure 14:**
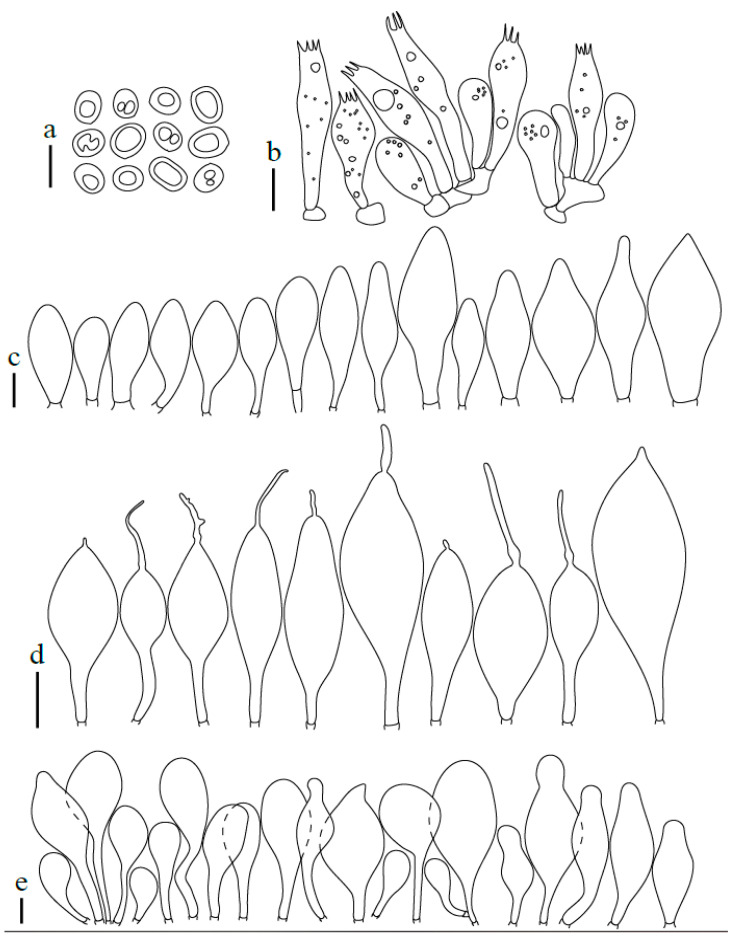
*Pluteus reisneri* OKA-TR1823: (**a**) spores; (**b**) basidia and basidioles; (**c**) pleurocystidia; (**d**) cheilocystidia; (**e**) pileipellis elements. Scale bars: 10 μm.

**Figure 15:**
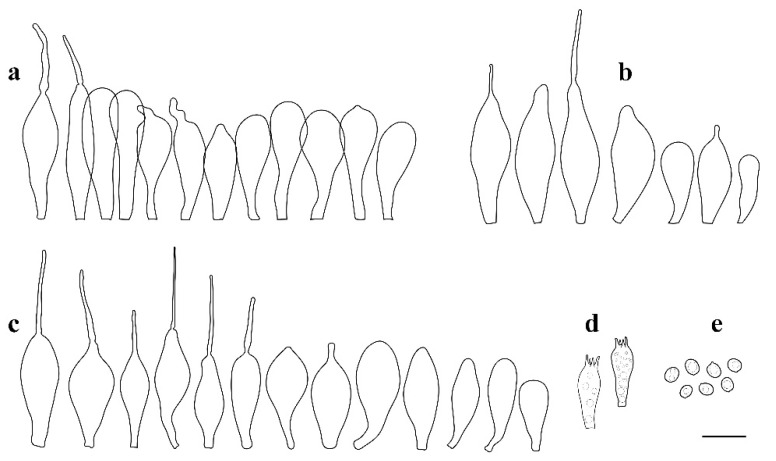
*Pluteus reisneri* FG14092019062-FG26092019042-FG29092019000: (**a**) pileipellis elements; (**b**) caulocystidia; (**c**) cheilocystidia. (**d**) basidia; (**e**) spores. Scale bar: 20 μm.

*Pluteus reisneri* Velen. (Figure 12, Figure 13, Figure 14 and Figure 15)

Holotype: České Houby 3: 610 [16] CZECH REPUBLIC: Slivenec, wet ravine, dead stem of *Rubus*, May 1918 leg. Velenovský J. et Reisner O. (PRC Velenovský herbarium, bottle 135!)

Epitype: CZECH REPUBLIC. Ochoz u Brna, Kulatý dub, a fallen, decaying trunk of a more than 300 years old *Quercus* covered with moss, 49.277N 16.731E, 25 August 2016, leg. H. Ševčíková (BRNM 781263, MycoBank: MBT10006377), designate here.

Type study:

Basidiospores [30/1/1] (5.0–)5.5–7.8(–8.2) × (4.4–)4.8–7.0(–7.2) µm, Lav = 6.6 µm, Wav = 5.9 µm, Q = (1.0–)1.1–1.3(–1.4), Q* = 1.2, mostly subglobose, some globose or broadly ellipsoid, thick-walled. Basidia 20–35 × 6.5–8.5 µm, narrowly clavate, four-spored, rarely two-spored. Pleurocystidia not found. Lamellar edge destroyed in some parts, but sterile in good condition parts. Cheilocystidia 21–44(–55) × 10–17(–24) µm, numerous, narrowly to broadly fusiform, narrowly to broadly clavate, inflated-fusiform, some with short pedicel, with or without needle like rostrum up to 20 µm at apex, hyaline, thin-walled. Pileipellis a hymeniderm, composed of sphaeropedunculate, broadly clavate or broadly fusiform elements, 33–51(–56) × 21–40(–44) µm; rare elongate, narrowly clavate to narrowly subfusiform elements 55–68(–72) × 14–24 µm present solitary, in few places forming clusters; hyaline or with pale brownish vacuolar pigment, thin-walled. Stipitipellis a cutis of cylindrical, hyaline, thin-walled, (6.2–)7.0–12.0 µm wide hyphae. Caulocystidia moderately abundant, solitary or in small clusters up to 10 caulocystidia, 20–55 × 10–25 µm, narrowly clavate to cylindrical, rarely subfusiform to broadly subfusiform, some with narrow, needle-like, 2–26 μm long rostrum at apex, thin-walled, hyaline. Clamp connections absent at all septa.

Summary Description of all Studied Collections

Pileus 13–41 mm diam., convex to planoconvex when young, then applanate to plano-concave, with or without distinct umbo; indistinctly wrinkled at the center, rarely distinctly veined, striate at margin up to one quarter to one half of pileus, rarely (Turkish collections) with a slightly reflexed and non-striate margin; surface dull, velvety-pruinose, not or weakly hygrophanous, brown-grey, brown, brightly brownish or grey (RAL 040 20 05, RAL 040 30 05, RAL 075 30 10–20, RAL 080 50 30–40, RAL 080 60 40, RAL 090 20–30 10, RAL 090 40 20–30), darker at the centre (RAL 70 20 10, RAL 080 30 05–10, RAL 080 20 05–10). Lamellae free, rather to moderately crowded, ventricose, up to four millmeters broad, whitish to cream, later pale pink to light dirty pink, with concolorous or white finely flocculose edges. Stipe 20–45 × 1.5–3.5 mm, cylindrical or curved, sometimes broadened at the base, but without basal bulb, longitudinally fibrillose, pruinose, white to dirty whitish or pale grey (RAL 050 90 05 RAL 060 30–40 05–10); often with indistinct white or grey-brown (concolorous with pileus) floccules. Context of pileus and stipe concolorous or slightly paler. Smell indistinct, taste not recorded.

Basidiospores [600/21/17] (5.3)5.5–8.0(–8.6) × (4.2)4.7–6.9 µm (Lav = 6.5 µm, Wav = 5.8 µm, Q = (1.0–)1.1–1.4(–1.6); Q* = 1.3, subglobose to broadly ellipsoid, rarely globose, thick-walled. Basidia 21–41 × 6–10 µm, 4-spored, narrowly to broadly clavate with central depression or subutriform. Pleurocystidia absent or rare, less frequently rather numerous, (22–)25–80 × (9.5–)11–34 µm, (sub)utriform, broadly clavate, broadly fusiform, with an obtuse apex or occasionally lageniform with a short neck, without rostrum at apex, hyaline, thin-walled. Lamellar edge sterile. Cheilocystidia 18.5–55(–59) × 6.5–18(–22) µm, numerous, clavate to broadly clavate, subfusiform, narrowly to broadly fusiform, rarely also broadly lageniform or utriform, some with short to long refractive rostrum at apex, rostrum 3–31(–45) × 1–2.2(–3) µm, some with a long pedicel up to 15 μm long, hyaline, thin-walled. Pileipellis a hymeniderm, made up of predominantly sphaeropedunculate with short to long neck up to 15(–25) µm long, some with a longer pedicel up to 32 μm long, vesiculose to clavate or narrowly to broadly fusiform elements or narrowly utriform to utriform, 20(–24)–41(–46) × (12–)14.5–28(–32) µm, without apical excrescence, very rarely also with rostrum up to 30 µm long, some with very rare longer cylindrical, narrowly clavate and/or subfusiform elements 48–63(–67) × (16–)18–26(–28) µm; all types with brown intracellular pigment and thin-walled. Stipitipellis a cutis of cylindrical, hyaline, thin-walled, 5–10(–12) µm wide hyphae. Caulocystidia numerous (absent in Turkish collections), in bundles, 15–42(–56) × (6)8–19 µm, clavate, narrowly fusiform to fusiform, rarely cylindrical, some with narrow to broad obtuse apex, some with short to long refractive apical rostrum, rostrum 2–35(–46) × 1–2.5(–3) µm, hyaline, thin-walled. Clamp connections absent at all septa.

Additional collections examined: CZECH REPUBLIC. Adamov, Býčí skála Nature Reserve, fallen trunk of *Fagus*, 49.305N 16.693E, 13 September 2018 leg. H. Ševčíková (BRNM808992); Brno–Bystrc, Jelení žlíbek Nature Reserve, fallen trunk of *Fagus*, 49.237N 16.485E, 17 June 2016 leg. H. Ševčíková (BRNM788196); fallen mosses trunk of *Fagus*, 49.237N 16.486E, 17 June 2016 leg. H. Ševčíková (BRNM825833); ibid., 1 August 2016 leg. H. Ševčíková et V. Antonín (BRNM792933); Bílá, Salajka Nature Reserve, fallen trunk of *Fagus*, 49.401N 18.419E, 7 August 2016 leg. V. Antonín et D. Janda (BRNM788198); ibid 25 August 2019 leg. V. Antonín et D. Janda (BRNM817757); ibid fallen mosses trunk of *Fagus*, 25 August 2019 leg. H. Ševčíková (BRNM817756); Karolinka, Smradlavá Nature Reserve, fallen trunk of *Fagus*, 49.401N 18.415E, 23 July 2018 leg. H. Ševčíková (BRNM808902); Ochoz u Brna, Kulatý dub, more than 300 years old fallen *Quercus*, 49.277N 16.731E, 2017, leg. H. Ševčíková (BRNM); Olomouc-Černovír, Černovírské slatiniště, floodplain forest with *Quercus robur* and *Fraxinus angustifolia*, with young *Ulmus*, deciduous stump, 49.625N 17.264E, 26 August 2019 leg. V. Halasů (BRNM825834); ESTONIA, Hiiu County, Kõrgessaare Commune, Sigala, swamp forest, 59.01062N, 22.55977E 19 August 2011, V. Liiv (TUF118320, initially identified as *Pluteus insidiosus*); SLOVENIA: Nova Goricȃ, Panoveĉ Park, on a gravel road on small buried woody shrubs in a mixed deciduous forest, 45.940N 13.677E, 14 September 2019 leg. G. Ferisin (FG14092019062); ibid., 26 September 2019 leg. G. Ferisin (FG26092019042); ibid., 29 September 2019 leg. G. Ferisin (FG29092019000); TURKEY. Bolu Province: Seven Lakes National Park, near Deringöl, on the wood of *Fagus orientalis*, at 40°56′35.4″ N, 31°44′57.4″ E, alt. 170 m, 1 November 2012, O. Kaygusuz, OKA-TR1823; ibid., at 40°56′35.6″ N, 31°44′57.0″ E, alt. 178 m, 03 November 2012, O. Kaygusuz (OKA-TR1824).

Notes: In the protologue [16], the stipe of *P. reisneri* was described as white, the pileus as grey-brown with a rugulose center and a striate margin, and it was stated to be growing on a *Rubus* sp. stem. Velenovský [16] mentioned subglobose basidiospores (7.0–8.0 µm long), but our type study found them to be slightly smaller. The pileipellis is formed by mostly sphaeropedunculate, broadly clavate or broadly fusiform elements, but also rare elongate elements up to about 70 µm long or rostrate elements are present. Cheilocystidia are rostrate, and caulocystidia are rostrate in some collections, while pleurocystidia were not found. The holotype is preserved in Velenovsky fluid (formaldehyde and ethanol solution [16,47]), and all attempts to sequence this more than 100 years old holotype have failed. To support an unambiguous identity of *P. reisneri*, we therefore designate an epitype based on collection from the Czech Republic, which fully corresponds to the original macroscopical description and microscopically match all features observed in the holotype.

Based on the holotype, epitype and several recent collections, *P. reisneri* is characterized by a medium to dark brown or grey, slightly pruinose pileus, which is usually striate at the margin. In some collections the pileus has been noted as somewhat velvety, but glabrous and neither pruinose nor velvety after rain. The stipe has indistinct dark or rarely whitish floccules, cheilocystidia and caulocystidia are frequently to moderate often rostrate and the pileipellis consist of predominantly sphaeropedunculate, vesiculose to clavate and fusiform elements without apical excrescence. Some collections (BRNM788196, BRNM825833) have also rare elongate elements in the pileipellis, similarly to *P. reisneri* holotype and epitype. Rare rostrate pileipellis elements around the pileus center are present in both Slovenian and some Czech collections (e.g., BRNM788196, 788198), while only one short appendix was found in the holotype, and also in the epitype collection. In most collections the pileus was indistinctly wrinkled at the center, but the distinctly wrinkled collection (BRNM781263) selected as epitype evokes the *P. reisneri* protologue. Velenovský in 1921 [16] mentioned the similarity of *P. reisneri* with *P. phlebophorus*. The epitype fully match this feature. The protologue of *P. reisneri* reported that the stipe is pruinose only on the upper part and the species is known only from *Rubus* sp. stem and in the moss among the grass [16]. The epitype (BRNM781263) was found on mosses decaying trunk of *Quercus*. No recent collection growing on *Rubus* sp. stem was found. It is rather distantly related to *P. insidiosus* and all other taxa treated in this paper, forming a sister clade to all these taxa and the *P. thomsonii* complex.

## 4. Discussion

The European specimens of *Pluteus* with a brown pileus, a whitish or silvery grey stipe, a pileipellis formed as a hymeniderm of sphaeropedunculate to narrowly clavate elements and some cheilocystidia with rostrum were traditionally identified as *Pluteus insidiosus* [3,12,48,49]. Without the molecular analysis support, it would be impossible to decide whether the subtle differences observed between individual collections represented one or more species, but with this study, we have revealed at least six species within the *P. insidiosus* broad sense from Eurasia. Several of these appear to be semicryptic, at least based on the current knowledge, while others are easier to differentiate. Macroscopically, typical basidiomata of *P. reisneri* clearly differ from all similar species by the combination of a slightly velvety-pruinose pileus and a stipe with delicate dark or rarely whitish floccules. However, glabrous basidiomata were also found, especially after rain. Furthermore, *P. farensis* may appear with an opaque, velvety or glabrous pileus. *Pluteus flavostipitatus* is macroscopically recognizable by a candle yellow stipe color, while *P. pseudoinsidiosus* differs from all similar species only when a greenish-blue stipe is present.

Microscopically, important characteristics usable for species delimitation are the shape and size of cheilocystidia and pleurocystidia, and also the presence and shape of caulocystidia. Rostrate cheilocystidia are a predominant feature for most species in this group, and their shape varies in being narrowly to broadly clavate, narrowly to broadly (sub)utriform, (sub)fusiform, rarely inflated-fusiform, oblong or ellipsoid. *Pluteus assimilatus* is only one species without rostrate cheilocystidia, while cheilocystidia in the single known collection of *P. flavostipitatus* presented only short rostra. The presence of a sterile or heterogenous lamellar edge does not appear to be a useful feature in separating taxa recognized here (see notes of *P. insidiosus*), while the shape and partly also size of pleurocystidia are significant characteristics. Unfortunately, pleurocystidia are very rare in most taxa, which reduces the usefulness of this characteristic for distinguishing these species. Caulocystidia in tufts are common in the entire stipe of *P. reisneri* and *P. assimilatus*, mostly only near apex in *P. farensis*, rare in *P. pseudoinsidiosus*; and usually lacking in *P. insidiosus* and *P. flavostipitatus*. Some rostrate caulocystidia are present in *P. reisneri* and *P. pseudoinsidiosus*. More collection of these species may verify more distinct differences between them.

Most of the species described here appear on decayed wood (stumps or trunks) of angiosperms, sometimes on wood covered with mosses, rarely on the wood debris, only *P. farensis* has terrestrial growth. *Pluteus reisneri* has been recorded also from mossy ground among grasses and from *Rubus* sp. stem. The biogeography of the treated species needs further study as, previously, most of these collections would have been identified as *Pluteus insidiosus*. Based on phylogenetically confirmed findings, the presumable distribution area of *P. flavostipitatus* is Far Eastern Russia, *P. assimilatus* may be limited to the territory of Northern Europe (Estonia and Northwestern Russia), *P. pseudoinsidiosus* is widespread, but rare from Central Europe across southern Europe to Western Asia (the Czech Republic, France, Spain and Iran) and *P. farensis* is so far known only from Italy.

The hotspot of *P. reisneri* seems to be Central Europe, especially the Czech Republic, but this species is also known in Slovenia and Turkey. Basidiomata of true *P. insidiosus* were collected and molecularly confirmed only from the Netherlands. However, data from Estonian soil samples show that *P. insidiosus* is also present in Estonia. Environmental DNA samples have also detected *P. assimilatus*, *P. farensis* and *P. pseudoinsidiosus*, broadening their known geographic distribution beyond the collection-based records. The distribution of the European species in this complex seems to be broad and overlapping to some degree.

The nuclear ribosomal internal transcribed spacer (nrITS) has been proposed as the universal barcode marker for fungi [50], but currently, a universal specific threshold value has not been recommended for *Pluteus* in previous studies. It seems likely that the traditional 97% similarity cut-off point is too broad to accurately separate *Pluteus* species in many instances. We do recommend the use of *TEF1-α* as a complementary barcode in *Pluteus*, especially when nrITS variation suggests the presence of more than one species within well-supported nrITS clades. Considering the “Clade I” which includes *P. insidiosus*, our results indicate that an nrITS patristic distance of 0.0134 (corresponding to nine different nucleotides including gaps in the whole region) is enough to separate two different species. The nrITS1 region is less variable than nrITS2, and patristic differences greater than 0.0082 can be considered as a possible threshold value in nrITS1, whereas this threshold value represents the maximum intraspecific value. The absence of conflicts between the nrITS and *TEF1-α* phylogenetic analyzes conducted separately confirms that this combination of markers is suitable for the separation of different phylospecies, as already reported in previous studies [51,52]. Conversely, the use of a combined nrITS and *TEF1-α* dataset in phylogenetic analyses do not accurately resolve the relationship between different species within “*Pluteus insidiosus* complex” suggesting that other markers will be needed to confirm these relationships.
Identification Key1 Cheilocystidia without rostrum…………………………………………………………………………………………………………………………………………………*Pluteus assimilatus*1 Cheilocystidia with rostrum……………………………………………………………………………………………………………………………………………………22 Stipe candle yellow (RAL 060-70-40)……………………………………………………………………………………………………………………………………………………*Pluteus flavostipitatus*2 Stipe whitish or pale greyish, sometimes with a bluish tinge………………………………………………………………………………………………………………………………………………33 Caulocystidia abundant, in clusters at least at lower part of the stipe, pileipellis without or with very rare elongate elements up to 70 µm, in few places in bigger clusters or not……………………………………………………………………………………………………………*Pluteus reisneri*3 Caulocystidia rare or absent at least at the lower part of the stipe, pileipellis without elongate elements………………………………………………………………………………………………………………………………………………44 Stipe with bluish to greenish tinge…………………………………………………………………………………………………………………………………………………*Pluteus pseudoinsidiosus*4 Stipe without bluish tinge…………………………………………………………………………………………………………………………………………………55 Basidiospores predominantly globose to subglobose, clavate caulocystidia present at the upper part of the stipe…………………………………………………………………………………………………………………………………………………*Pluteus farensis*5 Basidiospores predominantly subglobose to broadly ellipsoid, caulocystidia absent or very rare…………………………………………………………………………………………………………………………………………………66 Pleurocystidia absent or very rare, without rostrum; lamellar edge heterogenous, in some parts with cheilocystidia forming clusters rendering the lamellar edge sterile, in other parts fertile with only scattered cheilocystidia…………………………………………………………………………………………………………………………………………*Pluteus insidiosus*6 Pleurocystidia absent or very rare, some with rostrum; lamellar edge sterile…………………………………………………………………………………………………………………………………………………*Pluteus pseudoinsidiosus*

## Figures and Tables

**Table 1:** *Pluteus* collections used in phylogenetic analyses. Sequences with an * were newly generated for this study.

Taxon	Collection	Country	ITS	TEF1
*P. phlebophorus*	AJ81 (NBM-F-009110)	Spain	HM562039	ON133554 *
*P. rugosidiscus*	BRNM761706	Slovakia	MH010876	LT991752
*P. “insidiosus”*	MCVE 15120	Italy	JF908626	–
*P. assimilatus*	TUF118809	Estonia	UDB019488	ON133546 *
*P. assimilatus*	Environmental sample	Estonia	UDB0108769	–
*P. assimilatus*	Environmental sample	Estonia	UDB0295281	–
*P. assimilatus*	Environmental sample	Estonia	UDB0431493	–
*P. assimilatus*	Environmental sample	Estonia	UDB0188089	–
*P. assimilatus*	LE313452!	Russia (North western FD)	ON118385 *	ON133545 *
*P.* cf. *thomsonii*	MCVE 603	Italy	JF908607	–
*P.* cf. *thomsonii*	LE234787	Russia	FJ774084	–
*P.* cf. *thomsonii*	LE303662	Russia (Siberia)	KX216329	–
*P.* cf. *thomsonii*	LE303685	Russia (Siberia)	KX216328	ON133555 *
*P.* cf. *thomsonii*	AJ206(LOU)	Spain	HM562053	–
*P.* cf. *thomsonii*	GM2976 (NBM)	Spain	ON118386 *	ON133556 *
*P.* cf. *thomsonii*	MA-Fungi 54629	Spain	HM562066	–
*P.* cf. *thomsonii*	iNaturalist 37478844	USA (California)	iNaturalist 37478844	–
*P.* cf. *thomsonii*	UC1859868	USA (California)	KF306027	–
*P.* cf. *thomsonii*	AMD 120 (SIU)	USA (Illinois)	HM562067	–
*P.* cf. *thomsonii*	Homola930 (MICH)	USA (Michigan)	HM562197	–
*P. dominicanus* var. *hyalinus*	SP393695	Brazil	FJ816665	–
*P. farensis*	Environmental sample	Estonia	UDB0642780	–
*P. farensis*	GDOR5086	Italy	ON118387 *	–
*P. farensis*	GDOR5085!	Italy	ON118388 *	–
*P. flavostipitatus*	LE 313350!	Russia (Far East)	ON118389 *	ON133542 *
*P. insidiosus*	Environmental sample	Estonia	UDB075094	–
*P. insidiosus*	Environmental sample	Estonia	UDB0528968	–
*P. insidiosus*	Environmental sample	Estonia	UDB0651839	–
*P. insidiosus*	L0053623!	Netherlands	ON118390 *	ON133541 *
*P. pseudoinsidiosus*	BRNM747560	Czech Republic	ON118391 *	ON133543 *
*P. pseudoinsidiosus*	Environmental sample	Estonia	UDB0240603	–
*P. pseudoinsidiosus*	Environmental sample	Estonia	UDB0282747	–
*P. pseudoinsidiosus*	Environmental sample	Estonia	UDB0436887	–
*P. pseudoinsidiosus*	Environmental sample	Estonia	UDB0616398	–
*P. pseudoinsidiosus*	Environmental sample	Estonia	UDB0225977	–
*P. pseudoinsidiosus*	LIP0401350!	France	ON118392 *	ON133544 *
*P. pseudoinsidiosus*	DMS10194422	Iran	ON118393 *	–
*P. pseudoinsidiosus*	GM3569	Spain	ON118394 *	–
*P. reisneri*	BRNM788198	Czech Republic	MN597451	–
*P. reisneri*	BRNM788196	Czech Republic	MN597450	–
*P. reisneri*	BRNM781263!	Czech Republic	LT838189	–
*P. reisneri*	BRNM792933	Czech Republic	ON118395 *	ON133553 *
*P. reisneri*	BRNM808902	Czech Republic	ON118396 *	ON133547 *
*P. reisneri*	BRNM817757	Czech Republic	ON118397 *	ON133550 *
*P. reisneri*	BRNM825834	Czech Republic	ON118398 *	ON133549 *
*P. reisneri*	BRNM817756	Czech Republic	ON118399 *	ON133548 *
*P. reisneri*	BRNM825833	Czech Republic	ON118400 *	ON133551 *
*P. reisneri*	BRNM808992	Czech Republic	ON118401 *	ON133552 *
*P. reisneri*	TUF118320	Estonia	UDB015599	–
*P. reisneri*	FG24062020015	Slovenia	ON118402 *	–
*P. reisneri*	FG29092019000	Slovenia	ON118404 *	–
*P. reisneri*	FG14092019062	Slovenia	ON118406 *	–
*P. reisneri*	FG26092019042	Slovenia	ON118405 *	–
*P. reisneri*	FG13102019047	Slovenia	ON118403 *	–
*P. reisneri*	OKATR1823	Turkey	OM654764 *	–
*P. reisneri*	OKATR1824	Turkey	OM654765 *	–
*Pluteus* sp.	AJ 606 (NBM-F-009111)	USA (New York)	KR022011	–
*Pluteus* sp.	AJ491 (NBM-F-009112)	USA (US Virgin Islands)	KM983712	–
Uncultured *Pluteus*	HATFD14-10	India	KU847900	–

## Data Availability

Publicly available datasets were analyzed in this study. This data can be found here: https://www.ncbi.nlm.nih.gov, accessed on 7 January 2021; https://www.mycobank.org, accessed on 5 April 2022.

## Supplementary Materials

The following supporting information can be downloaded at: https://www.mdpi.com/article/10.3390/jof8060623/s1, Figure S1: Best tree from the ML analysis of the 5.8S+nrITS2+TEF1-á-exone dataset. Bootstrap values ≥ 70% and posterior probabilities ≥ 0.90 are indicated on or below the branches.
