# Supplementary material for: Pluteus insidiosus Complex, Four New Species Described and Pluteus reisneri Resurrected
Source: J Fungi (Basel). 2022 Jun 10;8(6):623. doi: 10.3390/jof8060623 (PMC9225660; doi:10.3390/jof8060623)
Supplement: Supplementary file 1 [file jof-08-00623-s001.zip › jof-1750079-supplementary.pdf]

Journal of Fungi

Additional Materials

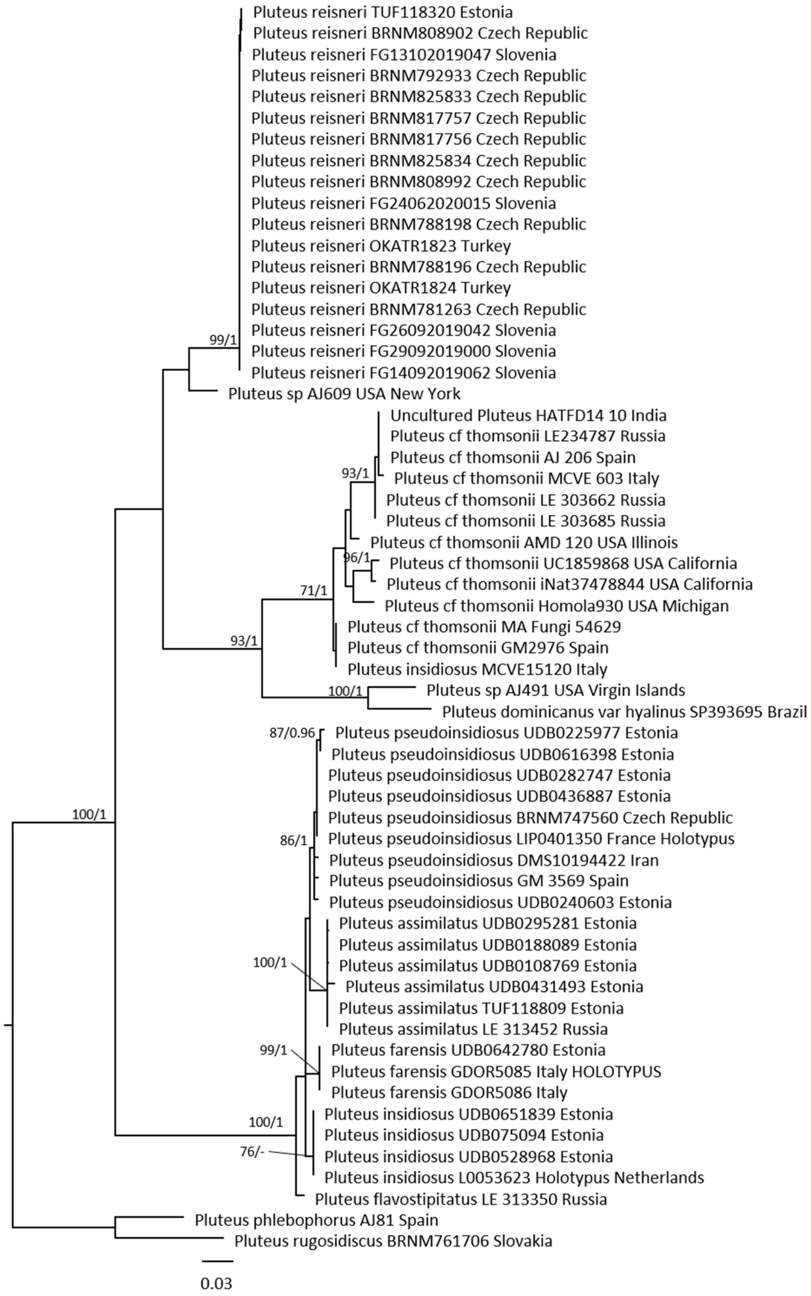

Figure S1. Best tree from the ML analysis of the 5.8S+nrITS2+*TEF1-α-exone* dataset. Bootstrap values ≥ 70% and posterior probabilities ≥ 0.90 are indicated on or below the branches.
